# The effect of concentrating obstetrics services in fewer hospitals on patient access: a simulation

**DOI:** 10.1186/s12942-016-0035-y

**Published:** 2016-01-22

**Authors:** Soichi Koike, Masatoshi Matsumoto, Hiroo Ide, Saori Kashima, Hidenao Atarashi, Hideo Yasunaga

**Affiliations:** Division of Health Policy and Management, Center for Community Medicine, Jichi Medical University, 3311-1 Yakushiji, Shimotsuke, Tochigi 329-0498 Japan; Department of Health Management and Policy, Graduate School of Medicine, The University of Tokyo, 7-3-1 Hongo, Bunkyo-ku, Tokyo, 113-0033 Japan; Department of Community-Based Medical System, Faculty of Medicine, Hiroshima University, 1-2-3 Kasumi, Minami-ku, Hiroshima, 734-8551 Japan; Department of Medical Community Network and Discharge, Chiba University Hospital, 1-8-1 Inohana, Chuo-ku, Chiba, 260-8677 Japan; Department of Public Health and Health Policy, Institute of Biomedical and Health Sciences, Hiroshima University, 1-2-3 Kasumi, Minami-ku, Hiroshima, 734-8551 Japan; Department of Planning, Information and Management, The University of Tokyo Hospital, The University of Tokyo, 7-3-1 Hongo, Bunkyo-ku, Tokyo, 113-8655 Japan; Department of Clinical Epidemiology and Health Economics, School of Public Health, The University of Tokyo, 7-3-1 Hongo, Bunkyo-ku, Tokyo, 113-0033 Japan

**Keywords:** Japan, Health policy, Obstetrical delivery, Hospital merger, Facility access

## Abstract

**Background:**

In Japan, the number of obstetrics facilities has steadily decreased and the selection and concentration of obstetrics facilities is progressing rapidly. Obstetrics services should be concentrated in fewer hospitals to improve quality of care and reduce the workload of obstetricians. However, the impact of this intensification of services on access to obstetrics hospitals is not known. We undertook a simulation to examine how the intensification of obstetrics services would affect access to hospitals based on a variety of scenarios, and the implications for health policy.

**Methods:**

The female population aged between 15 and 49 living within a 30-min drive of an obstetrics hospital was calculated using a Geographic Information System for three possible intensification scenarios: Scenario 1 retained facilities with a higher volume of deliveries without considering the geographic boundaries of Medical Service Areas (MSAs, zones of healthcare administration and management); Scenario 2 prioritized retaining at least one hospital in each MSA and then retained higher delivery volume institutions, while Scenario 3 retained facilities to maximize population coverage using location-allocation modeling. We also assessed the impact of concentrating services in academic hospitals and specialist perinatal medical centers (PMCs) alone.

**Results:**

In 2011, 95.0 % of women aged 15–49 years lived within a 30-min drive of one of 1075 obstetrics hospitals. This would fall to 82.7 % if obstetrics services were intensified into academic hospitals and general and regional PMCs. If 55.0 % of institutions provided obstetrics services, the coverage would be 87.6 % in Scenario 1, whereas intensification based on access would achieve over 90.5 % coverage in Scenario 2 and 93.9 % in Scenario 3.

**Conclusions:**

Intensification of obstetrics facilities impairs access, but a greater caseload and better staffing have the potential advantages of better clinical outcomes and reduced costs. It is essential to consult residents of hospital catchment areas when reorganizing clinical services; a simulation is a useful means of informing these important discussions.

## Background

In healthcare the correct balance between quality, cost and access are difficult to achieve, and provision of clinical obstetrics services is no exception. Several studies have demonstrated the effects of closure on hospital cost [1] and the efficiency of the hospital market [2]. Other studies have examined the effect of hospital closure on neonatal and infant mortality [3, 4]. A study of maternal deaths occurring in Japan suggested that the intensification of obstetrics services to avoid single-obstetrician facilities reduces maternal mortality [5]. Even if closure of hospitals can achieve better quality of care as well as reducing the burden on obstetricians and midwives, public opinion is often firmly against hospital closures because of the impact on access to healthcare services. Reducing hospital services has political implications that may override technical considerations [6]. Reorganization of maternity hospital supply is a highly sensitive topic and a matter for lively public debate [7, 8]. It is therefore important to be able to demonstrate improvements in outcome to balance against any perceived negative impact on access when services are reorganized. A simulation study of predicted outcomes after hospital closure conducted in the Netherlands found that the best strategy to avoid potential increases in intrapartum and first-week mortality was to close the ten smallest hospitals (approximately 10 % of the total number of hospitals), but to avoid closing adjacent small facilities [9]. Another study in Japan found a relationship between the time taken to drive to a specialist perinatal medical center and neonatal mortality rate, and concluded that neonatal mortality rate could be reduced by improving geographic accessibility to perinatal services [10].

In Japan, the number of obstetrics facilities has steadily decreased, and the selection and concentration of obstetrics facilities is progressing rapidly and effectively [11]. In 1996, there were 1720 hospitals and 52,976 deliveries, but these had fallen to 1126 hospitals and 47,626 deliveries by 2008 [12], representing a 3.5 % annual decline in the number of facilities and a 3.6 % increase in the rate of deliveries per hospital. This decline is considered to have occurred at least partly as a result of a shortage of obstetricians. As in many other more developed countries, obstetricians in Japan are reportedly demoralized by the fear of litigation and criminal negligence charges, and are leaving the profession [13]. An increasing proportion of female and younger obstetricians [14], who tend to put more emphasis on working fewer hours, life outside work and income [15], has also been identified as a factor contributing to the shortage of obstetricians. The disproportionate distribution of physicians in urban areas has been accelerating [16]. Consequently, preserving some hospitals in more rural areas does not necessarily mean that the hospital could function with sufficient staff.

Recently, the Medical Reform Committee of the Japan Society of Obstetrics and Gynecology published Grand Design 2015 (GD2015) entitled ‘Renovation of the obstetrics and gynecology healthcare system in Japan’ [17]. Although GD2015 did not set a target year, it recommended that services should be concentrated in regional flagship hospitals to reduce the burden on individual obstetricians and achieve sufficient numbers of full-time obstetricians in the core institution to populate a rotating shift system. It also recommended that local flagship obstetrics centers should be selected from existing secondary and tertiary public hospitals to provide a sustainable working environment for obstetricians.

The purpose of this study was to assess to what extent the negative effect on accessibility could be alleviated by different methods of service intensification. We defined optimal access to an obstetrics facility as the proportion of women of childbearing age (15–49 years) within a 30-min drive of a hospital providing obstetrics services. We considered intensification into academic hospitals and perinatal medical centers only, and three other scenarios: (1) consider only the number of deliveries (hospital volume) so as to maximize quality and minimize cost; (2) consider only maximizing accessibility; and (3) a combination of (1) and (2).

## Methods

### Setting

At the end of October 2011 there were 1896 municipalities forming 47 prefectures in Japan. The healthcare provision for several municipalities, divided into Medical Service Areas (MSAs) defined by Medical Service Law, is managed as a single unit of medical service provision for most diseases and conditions. Each prefecture regulates the number of hospital beds available in each MSA, overseeing the allocation of healthcare resources to provide services. In 2011, there were approximately 350 MSAs.

Medical services in Japan, including obstetrics services, are provided by hospitals or clinics. In Japan, medical facilities with 20 or more inpatient beds are defined as hospitals, and those with fewer than 20 as clinics. However, not all hospitals provide obstetrics services. We defined an obstetrics hospital as one that had an obstetrics department together with other support specialties. Among obstetrics hospitals, core facilities are selected and designated as perinatal medical centers (PMCs) by the government. There are two types of PMC. General PMCs have a maternal and fetal intensive care unit and accept round-the-clock emergency referrals of critically ill mothers and babies and high-risk pregnancies. Regional PMCs offer a higher level of specialist obstetric, neonatal and pediatric services [18]. In 2011, there were 8605 hospitals and 99,547 clinics in Japan; of these, 1075 hospitals and 46,386 clinics provided obstetrics services [19] and there were 100 general and 292 regional PMCs.

In Japan, cars may be driven by those aged 18 years or over, and lightweight motorcycles by those 16 years or older. Mean car ownership per household is 1.081 [20] and emergency transportation is provided free of charge.

### Data

We analyzed data from the most recently published Survey of Medical Institutions (Static Survey), undertaken in 2011. This survey is conducted by the Japanese government every 3 years, and covers all hospitals and clinics and is not limited to obstetrics facilities. Data are collected on clinical facilities and specific equipment available, staffing levels and services provided, but data concerning cost or outcome are not recorded. We obtained permission from the government to use individual hospital data for research purposes. Institutional data were current as of October 1, 2011 and the number of deliveries was recorded for the month of September 2011. Because of the effects of the Great East Japan earthquake, some of the affected areas were excluded from the survey and so were also excluded from our analysis. Population data were obtained from the 2010 National Census.

### Data analysis

To analyze the characteristics of the study subjects, obstetrics hospitals were classified into general PMCs, regional PMCs and others. Three scenarios were used to simulate the intensification of obstetrics hospitals based on the number of deliveries per hospital and the individual characteristics of the MSAs: Scenario 1, to retain higher volume (number of deliveries) obstetrics hospitals until the target number of hospitals was reached without considering the MSAs; Scenario 2, to retain higher volume obstetrics hospitals until the target number of hospitals was reached but retaining at least one obstetrics hospital in each MSA; Scenario 3, to retain obstetrics hospitals to maximize the proportion of the female population aged 15–49 residing within a 30-min drive of the nearest facility (Table 1).

**Table 1:** Three scenarios for intensification of obstetrics hospitals adopted in this study

Intensification scenario	Factor(s) to be considered		
	Hospital volume (number of deliveries per hospital)	Borders of Medical Service Area (MSA)	Population coverage
Scenario 1: Retain facilities with higher volume of deliveries per hospital without considering the geographic boundaries of MSAs	Yes	No	No
Scenario 2: Priority was first given to retaining at least one hospital in each MSA and then higher volume of deliveries per hospital	Yes (secondary)	Yes (primary)	No
Scenario 3: Keep facilities to maximize population coverage without considering the number of deliveries per hospital or MSAs	No	No	Yes

We selected three intensification targets for our simulations. Our selection was informed by the number of obstetrics facilities in 2011 (1075) and the number of PMCs and academic hospitals (405). We would have chosen targets of 1000, 800 and 600 hospitals to lie within this range, but revised these to take into account the proportion that did not submit data to the 2011 Medical Institution (Static) Survey because of the Great East Japan Earthquake. We therefore used adjusted targets of 985, 788 and 591. We reduced each target by 98.5 %, as in 2010—the year before the Earthquake—the number of hospital births in reported areas was 98.5 % of the national total [21].

In each scenario and for each target level of intensification, we calculated the number of deliveries, and the number of obstetricians and midwives reallocated to other institutions, and the national number of deliveries after intensification. Then, proportions of the female population aged 15–49 able to access the nearest obstetrics hospital within 30 min were calculated for each municipality, and the extent of the inter-municipality distribution difference was calculated using the Gini coefficient.

To assess any maldistribution caused by each scenario, the Gini coefficient was calculated to establish the level of inequity of the distribution of obstetrics facilities. The Gini coefficient is an index of unequal distribution widely used to assess inequity in incomes, but is now also used to examine healthcare resource distribution [22–26]. The index has a value between 0 and 1: when the distribution is totally unequal the value is 1, and exactly equal distribution is represented by a value of 0.

Analysis of variance was used to examine differences between group means. SPSS Statistics software (version 20.0J, SPSS IBM Japan Inc., Tokyo, Japan) was used for all statistical analyses except for the calculation of the Gini coefficient, for which Stata (release 12; StataCorp, College Station, TX, USA) was used. A *P* value <0.05 was considered statistically significant. The ethics committee of the Graduate School of Medicine and Faculty of Medicine, The University of Tokyo assessed and approved conduct of the study.

### Geographic Information System analysis

Based on our three scenarios and the three target levels for facility intensification, a 1-km^2^ grid was used to calculate the driving distance to the nearest obstetrics hospital using a Geographic Information System (GIS). Each square was classified as ≤30 min access, 30–60 min access or >60 min access. Then, the proportion of women aged 15–49 years resident in each 1-km^2^ section was calculated by taking the mean pixel value within each 1-km^2^ square and accumulated for each municipality, allowing us to make an assessment of the proportion of women of childbearing age living within 30 min of the nearest obstetrics facility. Each 1-km^2^ mesh within a 30-min drive was colored brown, within a 30–60-min drive was red and further than a 60-min drive was orange. Non-residential areas and non-reported areas were colored gray. The color scheme was chosen with reference to the ColorBrewer system [27].

We used MarketPlanner GIS (version 3.3.3, PASCO, Tokyo, Japan) for geographic analyses except for the selection of obstetrics hospitals in Scenario 3. MarketPlanner GIS version 3.2.6 with road network data version 2013 was used to estimate access time. Its estimation is based on actual travel speed or predefined speed based on the classification of the road (10–80 km/h) according to the software’s proprietary database.

In scenario 3, location-allocation analysis was undertaken to decide which hospitals should be retained using ArcGIS software (version 10.0, ESRI Japan, Tokyo, Japan). Location-allocation analysis is a tool in the ArcGIS Network Analyst extension that can determine the optimal locations for facilities to maximize coverage of the surrounding population [28] so that they can be allocated most efficiently [29].

## Results

### Status of obstetrics hospitals in Japan as of 2011

**Fig. 1:**
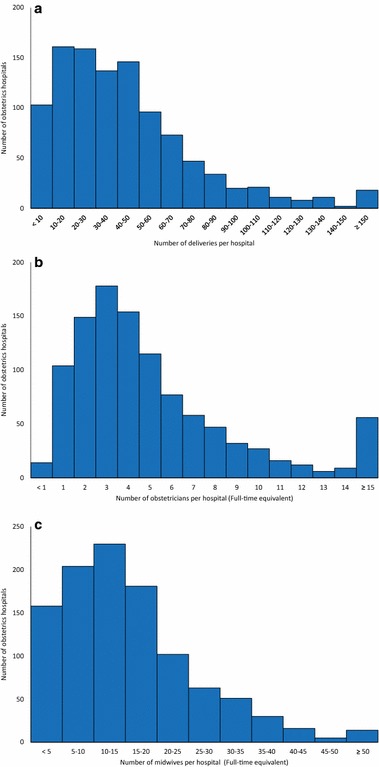
Distribution of the number of deliveries and staff per obstetrics hospital. Frequency distribution of the number of deliveries (**a**), obstetricians (**b**) and midwives (**c**) staff per hospital per month as of September 1–20, 2011 is shown. Staff numbers per hospital are represented as full time equivalents

**Fig. 2:**
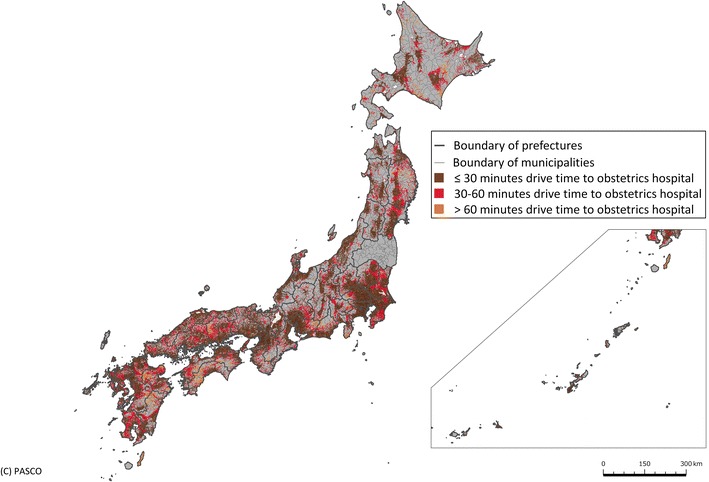
Access to obstetrics hospitals. Access to the nearest obstetrics hospital as of 2011. In each 1-km^2^ mesh, *brown* represents a driving time of ≤30 min, *red* a time of 30–60 min, *orange* a time of >60 min and *gray* represents non-residential and non-reported areas

**Fig. 3:**
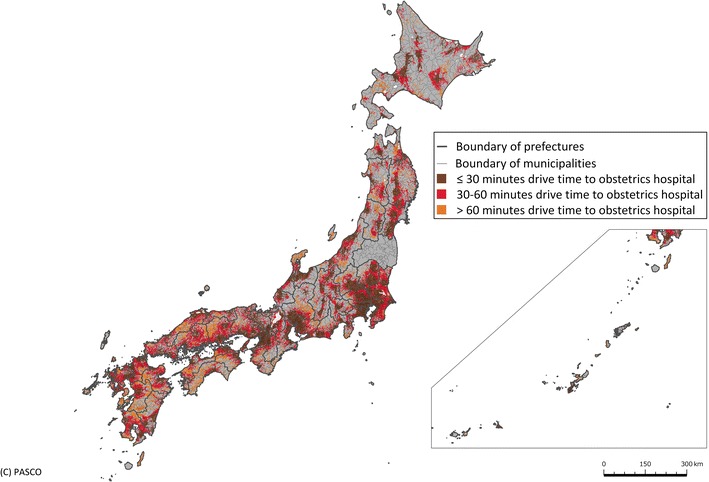
Access to academic hospital obstetrics departments or perinatal medical centers. Access to the nearest general or regional perinatal medical center or academic obstetrics hospital as of 2011. In each 1-km^2^ mesh, *brown* represents a driving time of ≤30 min, *red* a time of 30–60 min, *orange* a time of >60 min and *gray* represents non-residential and non-reported areas

Of the 1075 study subject obstetrics hospitals, the 95 general PMCs and 279 regional PMCs had higher numbers of staff and deliveries than the 701 other hospitals. The regional and general PMCs also had a lower number of deliveries per obstetrician and a greater proportion of deliveries by cesarean section (Table 2). Distribution of hospital volume (number of delivery per hospitals per month) and staff level (obstetricians and midwives) of obstetrics hospitals were skewed to the right (Fig. 1). Graphical presentations of access status to current obstetrics hospitals (n = 1075, Fig. 2) and academic hospitals and PMCs (n = 405, Fig. 3) are shown. Of the 15–49 year-old female population, 95.0 % currently have access to an obstetrics hospital within a 30-min drive, with 82.7 % having access to an academic hospital or PMC.

**Table 2:** Characteristics of institutions included in the study

	All hospitals providing obstetrics services (n = 1075)	Obstetrics hospital type			*P* value
		General perinatal medical centers (n = 95)	Regional perinatal medical centers (n = 279)	Other obstetrics hospitals (n = 701)	
Total hospital beds^a^ (SD)	380.5 (252.2)	738.6 (276.9)	518.3 (212.8)	277.0 (183.4)	<0.001
Obstetricians, number (SD)	5.5 (4.6)	12.3 (6.9)	7.0 (4.7)	4.0 (2.7)	<0.001
Midwives, number (SD)	15.3 (11.8)	31.5 (15.4)	20.0 (12.0)	11.3 (7.9)	<0.001
Deliveries per month, number (SD)	44.1 (39.3)	75.7 (59.8)	50.5 (38.7)	37.3 (32.8)	<0.001
Cesarean sections per month, number (SD)	11.3 (11.0)	26.2 (18.2)	14.5 (10.8)	7.7 (6.4)	<0.001

Data are presented as the mean (standard deviation, SD)

^a^Total number of hospital beds including non-obstetrics beds

### Intensification of obstetrics hospitals and access to obstetrics hospitals

**Fig. 4:**
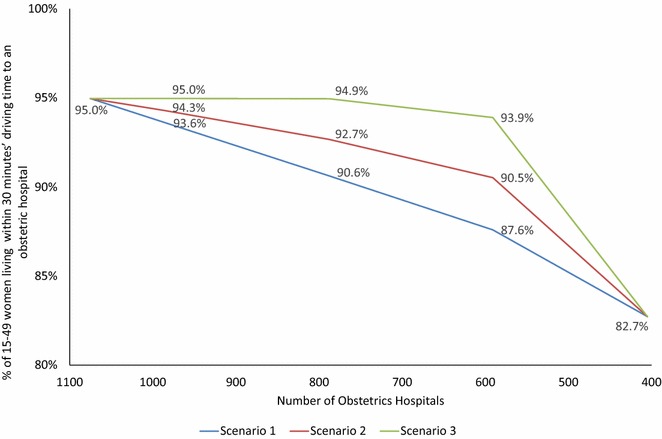
Extent of intensification and coverage of the female population of reproductive age. Intensification of obstetrics hospitals, access to the nearest obstetrics hospital and population coverage. Change in population coverage within 30-min access of the nearest obstetrics hospital based on three possible scenarios for intensification of obstetrics hospitals: Scenario 1 considered only the number of deliveries per hospital; Scenario 2 gave priority to retaining at least one hospital in each medical service area (MSA) and the number of deliveries per hospital in each MSA was considered; Scenario 3 aimed to maximize population coverage without considering the number of deliveries per hospital

The effects of intensification on hospital volume and staff level were estimated at the target levels of 985, 788, 591 (representing a national estimate of 1000, 800 and 600 obstetrics hospitals). For each target level of intensification, the number of deliveries and staff levels of obstetricians and midwives per hospital were the same for each scenario after intensification, but the number of deliveries and the number of staff that would need to be absorbed by retained institutions were larger when intensification emphasized hospital volume (Table 3). With regard to population coverage and inequity among municipalities, if intensification occurred without considering the MSAs (Scenario 1), access would fall in an indirectly proportional relationship. However, when MSAs were taken into account, impaired access could be avoided until intensification to 591 obstetrics hospitals (55.0 % from the 2011 level, equivalent to a national estimate of 600). At this level of intensification, coverage was calculated to be 87.6 % for Scenario 1, compared with 90.5 % for Scenario 2 and 93.9 % for Scenario 3 (Fig. 4).

**Table 3:** Estimated hospital volume after intensification

Number of obstetrics hospitals (national estimate)	985 (1000)	788 (800)	591 (600)	405
Number of hospitals to be absorbed	90	287	484	670
% of obstetrics hospitals in 2011 (1075)	92	73	55	38
No. of births/hospital after intensification	47.7	58.8	78.3	114.3
No. of obstetricians/hospital after intensification	5.9	7.3	9.8	14.3
No. of midwives/hospital after intensification	16.4	20.5	27.3	39.9
Scenario 1				
No. of births absorbed by other hospitals	280	3317	9984	24,213
No. of obstetricians in closed hospitals	164.6	699.7	1451.9	2420.0
No. of midwives in closed hospitals	461.7	2196	4485.8	7346.0
Scenario 2				
No. of births absorbed by other hospitals	414	4165	12,662	24,213
No. of obstetricians in closed hospitals	174.7	774.4	1644.1	2420.0
No. of midwives in closed hospitals	475.1	2284.2	4998.3	7346.0
Scenario 3				
No. of births absorbed by other hospitals	3336	12,724	18,254	24,213
No. of obstetricians in closed hospitals	383.0	1247.8	1825.1	2420.0
No. of midwives in closed hospitals	1001.2	3486.3	5287.7	7346.0

**Fig. 5:**
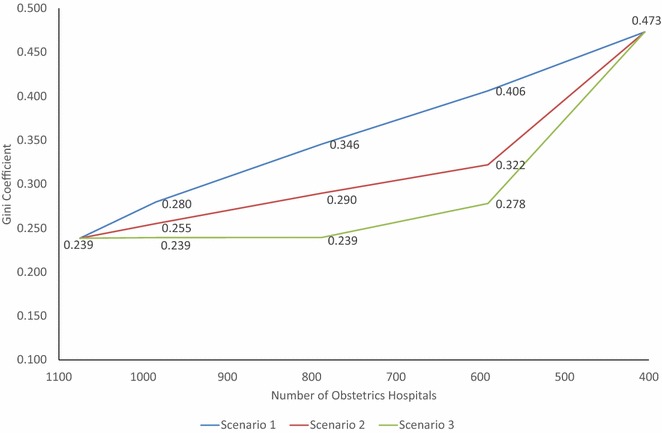
Extent of inequity between municipalities assessed by the Gini coefficient. Intensification of obstetrics hospitals, access to the nearest obstetrics hospital and level of inequity between municipalities. Inequity in 30-min driving-time access to obstetrics hospitals was calculated for municipalities using the Gini coefficient. In this model, the same three intensification scenarios were used to intensify towards 405 obstetrics hospitals

The Gini coefficient of 0.239 would rise to 0.473 when hospitals were intensified to PMCs and academic hospitals, indicating that the level of inequity would widen. Scenario 1 showed a directly proportional increase, but Scenarios 2 and 3 demonstrated a slower pace of increase in the Gini coefficient at intensification from the current level to academic hospitals and PMCs only, meaning that a greater extent of inequity can be avoided when MSAs and access are both taken into account (Fig. 5).

## Discussion

Although there is a trade-off between intensification of facilities and access to hospitals, the negative effect of intensification could be alleviated to a certain extent by considering the distribution of obstetrics facilities rather than simply considering hospital volume. However, striking the right balance between hospital volume and access is critical. On one hand, intensification by hospital volume alone will bring more deliveries and more medical staff to the retained hospitals, which will likely improve the quality of obstetrics services and reduce staff workload. On the other hand, too much emphasis on hospital volume will likely impair service delivery, particularly in already underserved areas, as those obstetrics hospitals located in resource-scarce (predominantly rural) areas tend to be lower volume and their closure creates a disproportionately greater impact on accessibility than closure of urban facilities. This is partly because when MSAs, designed to combine several municipalities into a self-contained service area, are taken into account in Scenario 2, we found that preservation of some low- and middle-volume rural hospitals maintains access while allowing intensification to almost half the current level.

Although this study was conducted in one country, our findings have global implications. Some studies have used GIS to simulate obstetrics facilities [30, 31], but their focus has been on access alone and not the influence of intensification of obstetrics facilities. Studies that have been undertaken in developing countries where obstetrics facilities may be scarce have mostly sought to identify means of increasing obstetrics coverage [32–35]. As the fertility rate declines in many more developed countries, the selection and concentration of obstetrics facilities will happen regardless of the number of obstetricians available to staff them. As the Japanese fertility rate is among the lowest in the world, our experience could provide a model for other more developed countries. Furthermore, as economic development matures, other less developed countries will ultimately face the same issues in the future. Our calculations of the numbers of deliveries and obstetrics staff that would need to be reallocated or redeployed according to each scenario, and modeling of different means of undertaking intensification, could inform public and political debate about the need for intensification of clinical services, which is a global issue. Healthcare policy makers, in Japan and other countries, must also consider means of incentivizing obstetricians to work in rural obstetrics facilities, to improve communication between hospitals, clinics and midwife-led services, and create healthcare management organizations that ensure optimal obstetrics care, considering not only the clinical services provided but also their accessibility.

Our study has several limitations. First, we employed driving time to define an outcome measure. This was estimated by GIS software, which does not take the traffic situation completely into account. Additionally, the clinical significance of longer driving times is not clear. A study of inter-facility neonatal transport found that neonatal mortality did not differ significantly between ≤30- and 30–60-min transfers [36]. Second, our intensification scenarios were based on the volume of deliveries as well as MSAs; however, management, administrative and cultural differences between organizations would likely influence the actual merger of hospitals. Reopening obstetrics hospitals or building obstetrics hospitals in new areas is another strategy that could improve access, but we did not take these factors into account.

## Conclusions

Closing hospitals or curtailing local obstetrics services is unpopular with the public. Policy makers planning intensification of obstetrics facilities must balance any negative impact on access against improved clinical outcomes and reduced costs. It is essential to consult residents of hospital catchment areas when reorganizing services. A simulation provides an evidence base to inform debates on what is frequently a highly controversial issue.

## Abbreviations

GIS: Geographic Information System
MSA: Medical Service Area
PMC: perinatal medical center
